# Targeted color design of silver–gold alloy nanoparticles†

**DOI:** 10.1039/d3na00856h

**Published:** 2023-12-07

**Authors:** N. E. Traoré, C. Spruck, A. Uihlein, L. Pflug, W. Peukert

**Affiliations:** a Institute of Particle Technology, Friedrich-Alexander-Universität Erlangen-Nürnberg Cauerstraße 4 91058 Erlangen Germany wolfgang.peukert@fau.de; b Interdisciplinary Center for Functional Particle Systems, Friedrich-Alexander-Universität Erlangen-Nürnberg Haberstraße 9a 91058 Erlangen Germany; c Department of Mathematics, Chair of Applied Mathematics (Continuous Optimization), Friedrich-Alexander-Universität Erlangen-Nürnberg Cauerstraße 11 91058 Erlangen Germany; d FAU Competence Unit for Scientific Computing (FAU CSC), Friedrich-Alexander-Universit, ä, t Erlangen-N, ü, rnberg Martensstraße 5a 91058 Erlangen Germany

## Abstract

This research article focuses on the targeted color design of silver–gold alloy nanoparticles (NPs), employing a multivariate optimization approach. NP synthesis involves interconnected process parameters, making independent variation challenging. Data-based property–process relationships are established to optimize optical properties effectively. We define a color target, employ a green chemical co-reduction method at room temperature and optimize process parameters accordingly. The CIEL*a*b* color space (Commission Internationale de l'Éclairage – International Commission on Illumination) and Euclidean distances facilitate accurate color matching to establish the property–process relationship. Concurrently, theoretical Mie calculations explore the structure–property relationship across particle sizes, concentrations, and molar gold contents. The theoretically optimal structure agrees very well with experimental particle structures at the property–process relationship's optimum. The data-driven property–process relationship provides valuable insights into the formation mechanism of a complex particle system, sheds light on the role of relevant process parameters and allows to evaluate the practically available property space. Model validation beyond the original grid demonstrates its robustness, yielding colors close to the target. Additionally, Design of Experiments (DoE) methods reduce experimental work by threefold with slight accuracy trade-offs. Our novel methodology for targeted color design demonstrates how data-based methods can be utilized alongside structure–property relationships to unravel property–process relationships in the design of complex nanoparticle systems and paves the way for future developments in targeted property design.

## Abstract 2

Mathematical, data-driven optimization of a green synthesis route for silver–gold alloy nanoparticles, controlling optical properties without a known formation mechanism.

## Graphical abstract

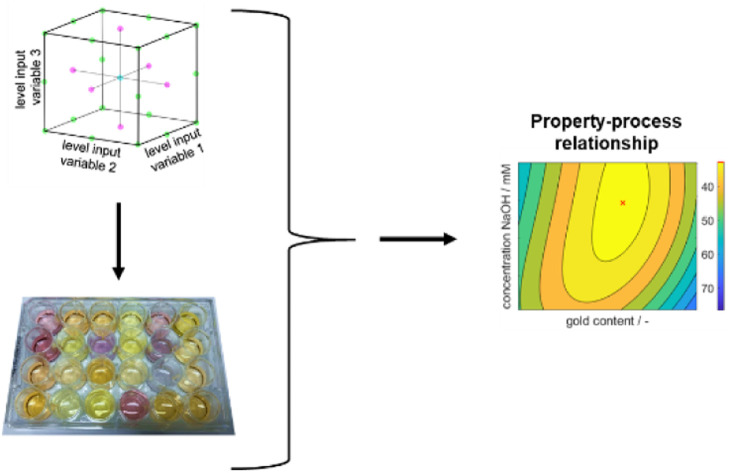

## 1 Introduction

Noble metal nanoparticles (NPs) have attracted significant attention due to their unique optical, electrical, and catalytic properties. Alloy NPs in particular consist of multiple materials with tunable composition-dependent properties with potential applications in nanosensing,^1^ antibacterial features^1,2^ and catalysis.^3,4^ Specifically, silver and gold are fully miscible and show the most pronounced localized surface plasmon resonances^5^ in optical applications.

Previous research has explored various methods for synthesizing silver–gold alloy NPs, predominantly in the liquid phase. These methods include laser syntheses,^6,7^ electrochemical reductions,^8^ biosyntheses,^9,10^ or most commonly, the chemical co-reduction of silver and gold precursors.^11–13^ The chemical co-reduction of silver and gold precursors involves the build-up of supersaturation of free silver and gold atoms *via* a reducing agent, *e.g.*, sodium borohydride^12^ or sodium citrate,^11^ followed by the formation of alloy NPs *via* nucleation and growth.^14–16^ We focused on a particularly “green” co-reduction method at room temperature, using dextran as both, the reducing agent and the stabilizer.^13^

The synthesis of bimetallic NPs involves the use of two different precursors. It is well established that the disperse properties of monometallic NPs are affected by the synthesis conditions, such as the temperature,^17^ pH^18,19^ or precursor concentrations.^20^ It is important to note that these conditions influence the conversion of both precursor materials in a different way. In fact, the synthesis conditions may favor the reduction, nucleation, or growth of one precursor over the other, leading to a final product with varying dispersity. This is especially challenging as the formation of silver–gold alloy NPs follows a highly complex multistage formation mechanism, which involves the formation of intermediates like solid silver chloride and core–shell particles.^13,21^

To optimize the properties of NPs, it is essential to understand the interdependencies between key process parameters and the resulting particle properties.^22^ Comprehensive mechanistic approaches can provide a detailed understanding of the underlying formation mechanism, making them the obvious choice for the knowledge-based derivation of property–process relationships.^14,16^ However, the data-based derivation of property–process relationships can be more efficient in highly complex, multi-component scenarios intractable for knowledge-based derivation. It is therefore often the method of choice in industry. This approach can provide insights into the effects of coupled synthesis parameters on NP properties and can further enable rapid optimization of the synthesis by identifying the most important process parameters.^23^

In complex systems, parameters are strongly coupled with each other and cannot be reasonably varied independently,^24^*e.g.*, the supersaturation as thermodynamic driving force is a highly nonlinear function of local precursor concentration. In most cases the resulting particles are not only distributed in size and shape but also their chemical composition. Therefore, testing the influence of different process conditions on the particle properties requires a multivariate parameter variation within the chosen parameter space. Multivariate parameter optimization for NP synthesis has been performed in literature before, including studies on the effect of the synthesis parameters within the Stöber process on the average size of the silica particles,^23^ the improvement of the dispersity of catalytically active silver NPs,^25^ the tuning of the properties of hexaferrite nanostructures^26^ and the optimization of the photoluminescence of CdSe quantum dots.^27^

In this study, we optimize the optical properties of silver–gold alloy NPs *via* a multivariate optimization approach. We produce the NPs by a previously published green chemical co-reduction method at room temperature, which allows for the highly reproducible synthesis of sub-10 nm particles with precisely adjustable chemical composition.^13^ The color of the final particle suspensions is chosen as the property to optimize by minimizing the distances of the produced colors to a color target in the CIEL*a*b* space. In addition we use our method to answer the question if a chosen color target is practically accessible by the synthesis method by clearly defining the area within the theoretically available color space that is practically accessible. To do that we simultaneously work with the property function and the process function and bridge both *via* our approach. Statistical experimental design then reduces the number of necessary experiments. Our study shows how property–process relationships for complex NP systems can be described in a data-based form with few experiments.

## 2 Materials and methods

### 2.1 Synthesis of silver–gold alloy nanoparticles

Silver nitrate solution (1 M) was procured from VWR Chemicals. Hydrogentetrachloroaurat(iii) trihydrate was obtained from Sigma Aldrich. Sodium hydroxide solution (1 M) was acquired from Honeywell. Dextran with a molecular weight of 40 kDa was purchased from ITW Reagents. All reagents were used as received without any further purification. For all experiments in this study, Millipore water with a resistivity of greater than 17 MΩ cm was used.

The NPs utilized in this research were synthesized according to the protocol outlined in.^13^ In short, a solution of dextran at different concentrations was prepared. 4.5 mL of this solution was transferred to a 10 mL snap cap vial and mixed with a variable volume (*x* mL) of an aqueous solution of AgNO_3_ and a variable volume [(1 − *x*) mL] of HAuCl_4_, also of different concentrations. Different compositions were produced by varying the value of *x* between 0 and 1. After mixing, 0.5 mL of an aqueous NaOH solution with different concentrations was added to the mixture to initiate the reduction process, followed by further mixing. The mixture was left overnight under the lit fume hood. After several hours, the dispersions displayed a range of colors from yellow to red, indicating the formation of stabilized silver/gold NPs.

### 2.2 Mie theory calculations

All Mie simulations have been carried out using STRATIFY,^28^ an open-source MATLAB implementation of a transfer matrix method for calculating near- and far-field electromagnetic properties of multilayered spheres. Mie calculations were employed to calculate the optical properties of particle suspensions with varying disperse properties. The refractive index data, obtained from literature,^29^ were interpolated on a more refined grid to account for different alloy compositions. Additionally, the dielectric function was adjusted to accommodate the small size of the alloy particles below 20 nm. The impact of small particle sizes on the electron behavior in the metal nanoparticles has been extensively discussed in previous studies.^30–33^ In summary, the reduced mean free path of electrons within the metal particles leads to an increase in collision frequency, impeding their relaxation. To address this phenomenon, the damping constant was modified according to *γ*_NP_ = *γ*_bulk_ + 2*Cv*_F_/*x*.^34^ Following the protocol established by Cardenas Lopez *et al.*,^35^ the values chosen for *C*_Ag_ = 0.2959 and *C*_Au_ = 0.2035 were determined. For alloy particles, a linear relationship between *C* and the molar gold content was assumed. A comprehensive comparison between the calculated and experimentally acquired optical spectra can be found in the ESI† of our recent publication.^13^ Mie theory was chosen for all optical simulations based on the strictly spherical shape of the NPs, which was confirmed in prior experiments (refer to Traoré *et al.*^13^).

### 2.3 Multivariate optimization

**Fig. 1:**
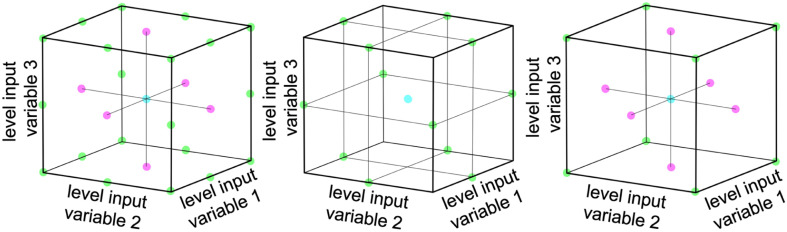
Comparison of measurement levels around a center point for a full factorial design (left), the Box–Behnken design (middle) and a face centered central composite design (right). Colored dots symbolize measurements.

Multivariate optimization is a technique used to optimize systems that depend on multiple variables. Three common designs for multivariate optimization are used within this work: full-factorial design, Box–Behnken design, and face centered central composite design.^36^Fig. 1 shows a graphical representation of each of the three design principles for a system with 3 input parameters.

A full-factorial design involves testing each possible combination of the chosen grid points. The number of experiments scales with *L*^*F*^, where *L* is the number of levels for each variable and *F* is the number of tested factors.^36^ The number of necessary experiments increases exponentially with an increase in the number of relevant factors. Therefore various schemes are employed to reduce the number of necessary experiments, while retaining the bulk of information.

The Box–Behnken design uses fewer experiments than a full-factorial design by leaving out the corners and the face center points of the parameter space. The parameter space is defined *via* concentric rings around the global center point.^37^ The number of experiments can be calculated as 2*F*(*F* − 1) + *P*_0_, where *F* is the number of tested factors and *P*_0_ is the number of center points.^38^ This design still captures quadratic relationships and is useful when the factors are continuous.^39^

The face centered central composite design consists of a two-level design, which is supplemented by additional face-centered points. It involves testing the extremes of the factors being optimized plus a few additional points and can therefore also capture quadratic relationships. The number of experiments can be calculated as 2^*F*^ + 2*F* + *P*_0_ where *F* is the number of tested factors and *P*_0_ is the number of center points.^38^ This design is useful when the factors are valid only in a defined range.^39,40^

### 2.4 Characterization

UV-vis spectroscopy was carried out using the Varian Cary 100 spectrophotometer in the wavelength range of 200 nm to 800 nm with a spectral resolution of 1 nm. A cuvette containing water as the solvent used in the synthesis was used as a reference to all samples. UV cuvettes with an optical path length of 1 cm were used for all measurements. Samples were taken directly from the synthesis, no further purification or dilution was performed.

Scanning transmission electron microscopy (STEM) images and STEM-based energy-dispersive X-ray spectroscopy (STEM-EDX) maps were taken in a Thermo Fisher Scientific Spectra 200 C-FEG microscope operated in STEM mode using a high-angle annular dark-field (HAADF) detector with a collection-angle of 56–200 mrad and an acceleration voltage of 200 kV. Before measurement, the NPs were washed with and redispersed in Millipore water and dried on a 200-mesh carbon-coated copper TEM grid (Plano GmbH). Subsequently the grid was cleaned in a plasma cleaner (Fischione Instruments Model 1070 NanoClean) using Argon plasma.

## 3 Results and discussion

### 3.1 Theoretically available color space

To optimize the synthesis of silver–gold (AgAu) alloy NPs according to our chosen route, we start with the optimization of the property function. To gain an understanding of the full color space for targeted optimization, we simulated the extinction spectra of silver–gold alloy NPs with varying molar gold contents (ranging from 0% to 100%), sizes (ranging from 4 nm to 40 nm), and particle number concentrations (ranging from 1.25 × 10^10^ mL^−1^ to 1.25 × 10^15^ mL^−1^ – covering a wide spectrum) using Mie theory. From the extinction data, we calculated the transmission spectra according to Beer–Lambert's law:

1 *T*(*λ*) = exp(−*cl*ext(*λ*))

where *T* is the wavelength dependent transmission, ext is the wavelength dependent extinction, *c* the particle number concentration and *l* the optical path-length of the measurement cuvette. These transmission spectra were then transformed into CIEL*a*b* coordinates using the tristimulus values *X*, *Y*, and *Z*:2
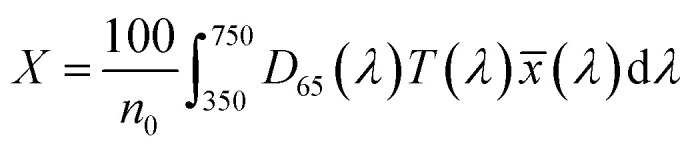
3
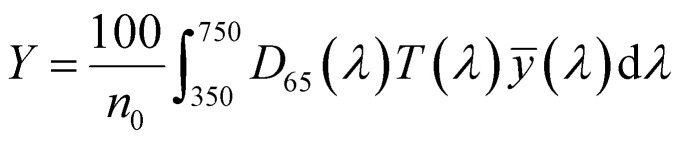
4
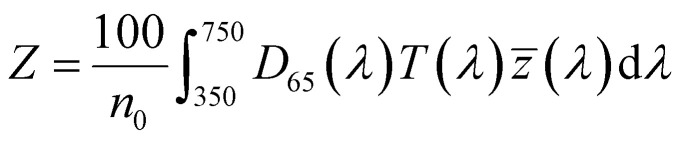
with:5
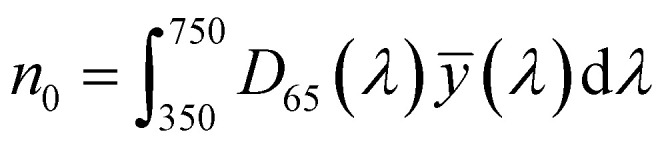
Here *x̄*, *ȳ* and *z̄* are the CIE 1964 standard color matching functions under a viewing angle of 10° to the color target (10° observer), which is commonly considered representative for how human eyes perceive color.^41^ The integral limits are the wavelengths of the equivalent monochromatic light in nanometers. To account for the color perception, we weighted the tristimulus values with reference white *D*_65_, corresponding to daylight.^42^ Finally, we derived the CIEL*a*b* coordinates *via*:6
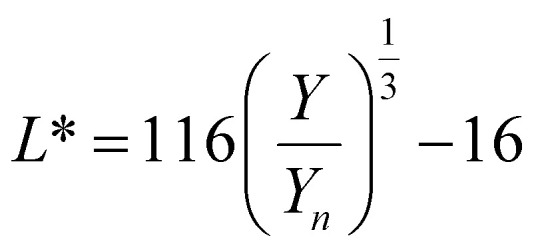
7
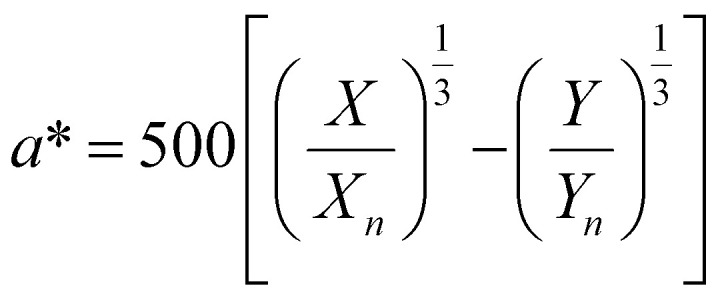
8
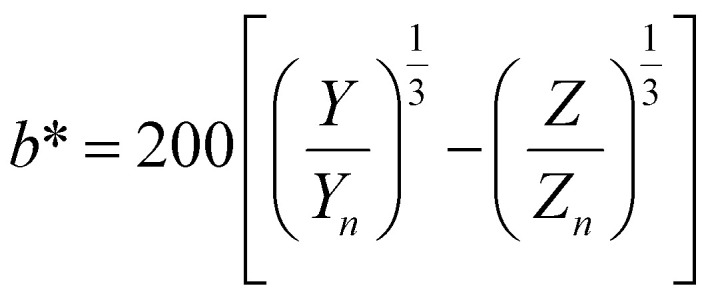
where *X*_*n*_, *Y*_*n*_ and *Z*_*n*_ are the tristimulus values of the reference white *D*_65_, with the values:9
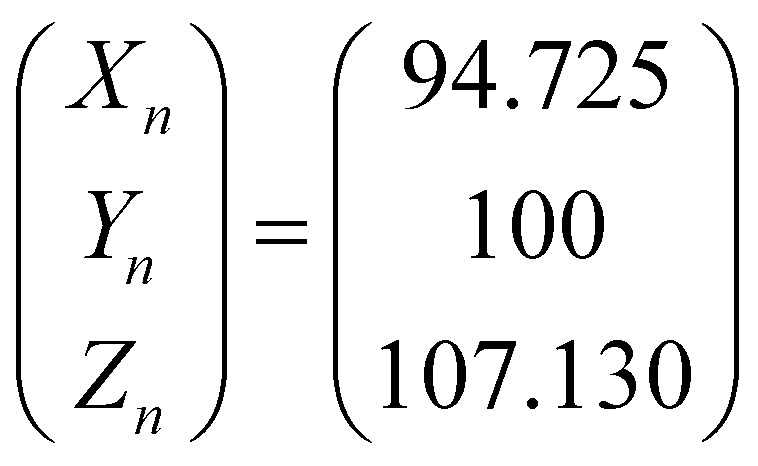


**Fig. 2:**
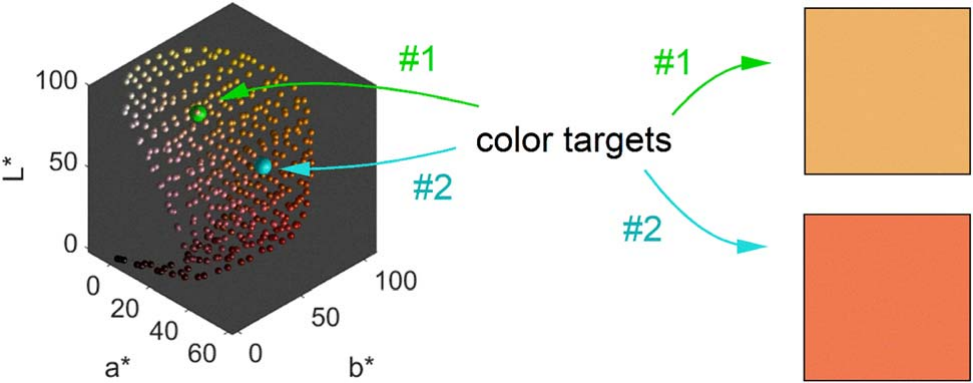
Representation of possible transmission colors of silver–gold alloy NPs with a molar gold content between 0 and 100%, a mean particle size between 4 and 40 nm and a particle number concentration between 1.25 × 10^10^ and 1.25 × 10^15^ mL^−1^. 2 chosen color targets for optimization are indicated in green and turquois and the color of the respective target is shown on the right.

We obtain a 3-dimensional color map (Fig. 2) that illustrates the range of possible colors. Consistent with existing literature, the color space covers a spectrum from bright yellow, characteristic of pure silver NPs, to red, typical of pure gold particles, with a smooth orange gradient in between. High particle concentrations resulted in a black color due to strong plasmonic NP absorption, while low particle concentrations exhibited a white color in the suspensions. These results align with prior findings.^11,13^

The main objective of this study is to optimize the color of a NP suspension. To achieve this, two color targets were chosen similar to the colors available in the color space and represented in the CIEL*a*b* space. The first target exhibits a high L* value and is similar to colors achieved *via* our synthesis route. As no model for the route exists, we assume this color with coordinates L* = 78, a* = 13, b* = 45 to be in the practically available color space (see green color target in Fig. 2). The second color target is freely chosen from the theoretically available color space (L* = 67, a* = 45, b* = 47) and has a significantly lower L* and higher a* value, which corresponds to a more saturated color with stronger red component (see turquois color target in Fig. 2).

To quantify the distance between colors in the color space and the defined color target, we calculated the Euclidean distance using eqn (10).10
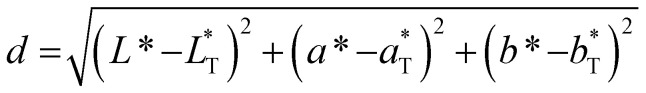


**Fig. 3:**
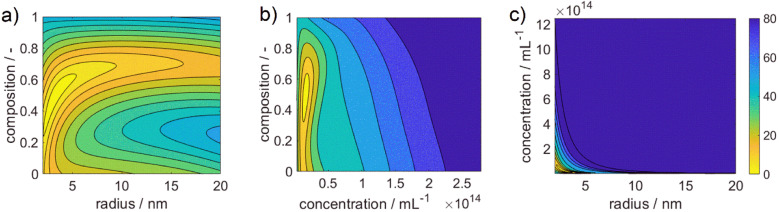
Plotted cost functional for the color difference of AgAu alloy NPs to the defined color target. The norm of the Euclidean distance in the CIEL*a*b* space is shown in the color pallet with brighter colors demonstrating smaller distances, *i.e.*, better agreement of color, and darker colors demonstrating larger distances.

Fig. 3 shows 3 cuts through the 4-dimensional function, describing the Euclidean distance to the first color target for various particle concentrations (b and c), sizes (a and c) and compositions (c). The function shows a clear optimum. The lowest deviations from the color target for specific NP characteristics are: particle diameter of 6.6 nm, molar gold content of 50%, and particle number concentration of 1.6 × 10^13^ mL^−1^.

The study establishes a structure–property relationship for the optical properties of the NP system. It is essential to consider that all calculations in this section are based on the assumption of stable monodisperse particles with a molar gold content. In reality, both of these properties are distributed around a modal value. Insufficient colloidal stability could lead to a change of optical properties of the NP suspension due to additional scattering effects.^35^ These distributions can also be taken into account in the model-based structure–property function. For spherical particles with distributed sizes and compositions, the presented procedure can still be applied.^43^ Dropping this assumption and considering anisotropic particles or even distributions thereof, impedes a full analysis of the admissible color range, but still allows for optimization of particle structure with respect to color.^44,45^ However, such settings require specialized optimization techniques.^46^

Moving forward, our approach involves working with experimentally produced NP suspensions with variations in size and chemical composition. We aim to transition from a theoretically derived structure–property relationship to a data-based process–property relationship to identify optimal process conditions for achieving the desired color.

### 3.2 Experimentally accessible color space

#### 3.2.1 Formation of silver–gold alloy NPs

**Fig. 4:**
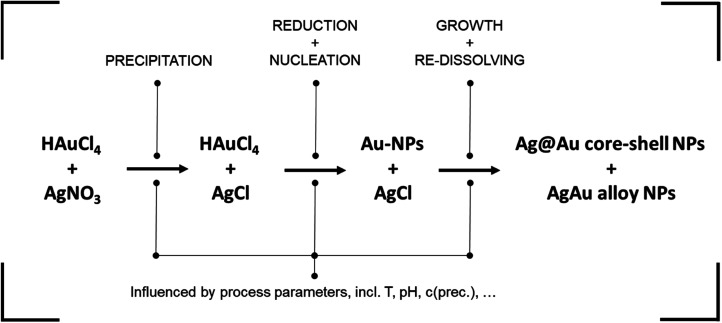
Schematic formation mechanism of AgAu alloy NPs according to the presented synthesis route. While the steps are shown in subsequent order, in reality all processes run simultaneously with dynamically changing reaction rates and are strongly interconnected.

We employed a reaction-controlled synthesis, known for its versatility across a wide range of concentrations.^13^ This method operates at ambient temperature and entails a chemical co-reduction process involving silver and gold precursors. Fig. 4 shows a schematic of the formation mechanism of the resulting AgAu NPs. AgNO_3_ is used as the silver precursor and HAuCl_4_ as the gold precursor. Dextran with a molecular weight of 40 kDa is used as the reducing agent as well as the stabilizer for the final particles. Through the silver precursor, free Ag^+^ ions are introduced into the reaction solution, whereas the gold precursor introduces free Cl^−^ ions. Consequently, due to the exceedingly low solubility of AgCl (solubility product *K*_sp,AgCl_ ≈ 10^−10^ mol^2^ L^−2^),^47^ solid AgCl precipitates upon the interaction of both precursors. This leads to a reduction in available Ag^+^ ions for subsequent reduction and eventual incorporation into the final NPs. As a result, the subsequent reduction of the metal precursor yields a surplus of gold atoms relative to the silver atoms.

Dextran assumes the role of the reducing agent, utilizing its terminal aldehyde group to facilitate the reduction of metal precursor ions through oxidation. The reduction process is notably contingent upon the pH of the reaction medium. Firstly, because the reducing aldehyde group reacts only in its deprotonated alkoxide form.^48^ This leads to an increase in reductive capacity and reaction rate at high pH values. Secondly, the pH impacts the formation of complexes between precursor ions and OH^−^ ions in solution, with distinct reactivity for silver^49^ and gold^20^ ions. This yields a complex dynamic behavior where increasing the solution pH increases silver reactivity while dampening gold reactivity.

In a next step, the built-up supersaturation of gold and silver atoms undergoes depletion through a crystallization reaction. Due to the notable excess of gold atoms, the nuclei formed initially exhibit a pronounced gold-rich composition. In essence, the initiation of particle nucleation is predominantly attributed to the gold precursor. As the reaction is reaction controlled with reaction times of up to 24 hours,^13^ AgCl re-dissolves over time while Ag is integrated into the formed NPs during their growth. Moreover, it is possible for the AgCl to be decomposed and directly reduced to atomic silver, mainly *via* light irradiation.^50^ In the process, a core–shell particle with a gold-rich core and a silver-rich shell can be expected, which transforms into a homogeneous alloy NP over time.

In summary, the presented NP synthesis presents a highly complex system, containing multiple chemical pathways as well as the formation of intermediate compounds. Synthesis parameters, such as the pH, are known to influence the reaction partners differently, adding to the complexity of the system. In lack of a mechanistic model, the complex system is suited to apply data-based methodologies for the derivation of property–process relationships. A detailed mechanistic study on the system followed by the derivation of the full knowledge-based property–process function is currently performed within our group.

#### 3.2.2 Experimental design

The synthesis, described in Section 3.2.1 is optimized using a multivariate approach. For that, we keep the reaction temperature, as well as the light irradiation into the system constant. Thus, the primary factors determining the dispersity of the resulting particles are as follows:

(1) The intended molar gold content of the NPs.

(2) The concentrations of the silver and gold precursors.

(3) The concentration of dextran, serving as the stabilizer and reducing agent.

(4) The concentration of NaOH, used to adjust the solution pH.

Preliminary experiments revealed that the dispersity strongly depends on the interplay of these parameters. Thus, merely varying individual parameters is insufficient to establish a property–process relationship for this particular system. Instead, we adopted a multivariate optimization approach, varying each parameter at 3 levels within their respective ranges. This strategy enables the identification of not only linear but also quadratic relationships among the parameters.

To comprehensively describe the chosen parameter space, 81 experiments were conducted (3 levels for each of the 4 parameters). The parameter range was refined in preliminary experiments to ensure stable particles with well-defined colors. Notably, while higher concentrations of the metal precursors are possible, the chosen particle system is a strong absorber, leading to ill-defined colors at high concentrations (see ESI Fig. S1†). Consequently, we excluded these high concentrations for the purpose of this study. Table 1 summarizes the selected parameter values for each level. It should be noted that additional factors can be taken into account by adding more parameters to the model, which results in an increased number of necessary experiments.

**Table 1:** Varied parameters and levels for the multivariate optimization approach. −1 represents the lowest and +1 the highest chosen value for the respective parameter. All parameters were linearly varied in the given ranges

Parameter/level	−1	0	+1
Molar gold content/—	0	0.5	1
Ag and Au concentration/mM	0.5	0.875	1.25
Dextran concentration/mM	0.00125	1.876	3.75
NaOH concentration/mM	5	102.5	200

#### 3.2.3 Property–process relationships

To gain insight into the impact of process-induced changes on particle dispersity and the resulting extinction color of particle suspensions, we look towards the process function. We defined a response variable, *i.e.*, the Euclidean distance between the color target and each measured value. The aim was to optimize this variable, with a smaller distance indicating a more desirable outcome. To calculate the distances, we transformed the measured spectra into CIEL*a*b* coordinates using eqn (2)–(8) and then fitted the L*, a*, and b* coordinates with a quadratic equation as a function of four variables:

11 L* ∨ a* ∨ b* = *i*_1_ + *i*_2_*N* + *i*_3_*D* + *i*_4_*M* + *i*_5_*G* + *i*_6_*N*^2^ + *i*_7_*ND* + *i*_8_*D*^2^ + *i*_9_*NM* + *i*_10_*DM* + *i*_11_*M*^2^ + *i*_12_*NG* + *i*_13_*DG* + *i*_14_*MG* + *i*_15_*G*^2^

where *D* stands for the concentration of dextran, *M* for the metal precursor concentration, *N* for the concentration of NaOH, *G* for the intended molar gold content of the resulting particles, and *i*_1_, …, *i*_15_ describe the respective pre-factors. The values of factors *i*_1_ to *i*_15_ for L*, a*, and b* can be found in the ESI (see Table S1†).

**Fig. 5:**
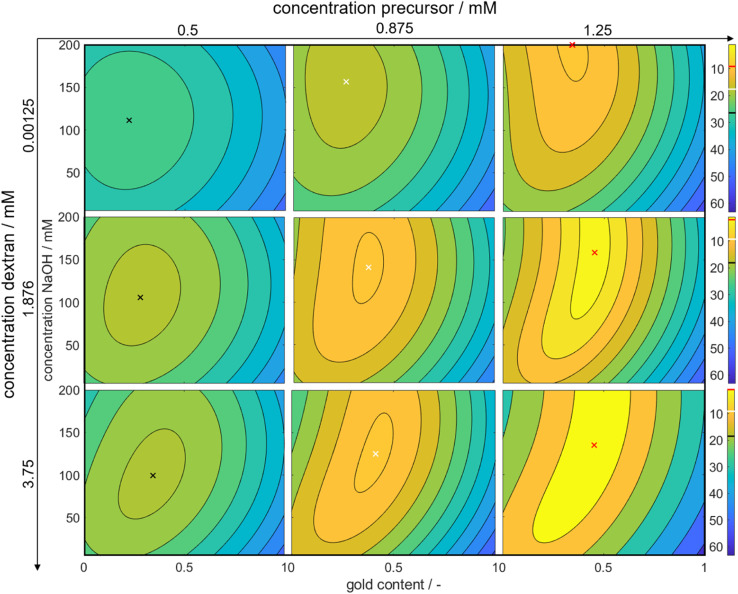
3-Dimensional plots of the Euclidean distances between the calculated CIEL*a*b* coordinates and the defined color target. Bright values symbolize optimal conditions, while darker colors symbolize larger distances to the color target. The exact values for the distances are shown in the color bars on the right of each line with the colored line showing the position of the local optimum within the subplot as marked by the cross in the respective color. The *y*-axes show the concentration of NaOH and the *x*-axes show the molar gold content. The concentration of metal precursor increases from the left to the right column of subplots, while the concentration of dextran increases from the top to the bottom row.

Through this approach, we can interpolate between the measurement points and access the predicted color at the optimal point. We plotted the Euclidean distances to the first color target for each of the calculated values as a heat map (see Fig. 5). The local optimum in each subplot is marked with a colored cross, and the distance value is marked with a line in the respective color on the color bars on the right. The full parameter space is 5-dimensional, only 3 dimensions are shown in the sub-plots with additional 2 in the columns (metal precursor concentration) and rows (dextran concentration) of the diagram. The *R*^2^ values of L*, a*, and b* are found as 74%, 89%, and 81%, respectively.

The heat map reveals clear optima for each subplot, with a prominent global maximum found for a molar gold content of 45%, a metal precursor concentration of 1.25 mM, a dextran concentration of 3.75 mM, and a NaOH concentration of 135 mM. The color target was more accurately matched with an increase in the metal precursor and dextran concentrations. This is due to an increase in extinction, which comes from an increase in added gold and silver mass. If more precursor is added to the system, more particles are formed, leading to an increased particle number concentration, finally leading to an increased extinction value according to Beer–Lambert's law. An increase in the metal precursor concentration requires an increase in pH and thus in NaOH concentration in order to circumvent the low pH of the gold precursor. Similarly, an increase in the dextran concentration requires a reduction in NaOH concentration. Both can be explained by the chemical structure of the reducing agent dextran, which, as a sugar predominantly reduces the metal salt in solution by oxidation of its terminal aldehyde group, which needs to be deprotonated to show reductive properties, as described above. Thus, the pH of the solution determines the reductive strength of the reducing agent *via* the percentage of deprotonated dextran in solution and needs to be adjusted accordingly. Consequently, the decrease in pH caused by the gold precursor needs to be compensated by the addition of more NaOH and an increase in the absolute number of reducing molecules needs to be compensated by a lower NaOH concentration.

Moreover, it is evident that an increase in the ratio of gold and silver precursor added to the system, requires a higher concentration of the reducing agent. This phenomenon can likely be again attributed to the higher reductive potential at higher dextran concentrations. The synthesis parameters are optimized to produce particles, whose suspension matches the color target as well as possible. As shown above, 3 factors are considered, *i.e.*, the particle number concentration, size and final composition. In reality all of those attributes are interconnected *via* the chosen synthesis conditions, meaning that a change in the conditions will influence all attributes, often in an antagonistic manner. Here, for a low dextran concentration the reductive potential is comparably low, leading to a lower optimal intended molar gold content. As we established that gold is predominantly responsible for the nucleation of the particles, if the amount of gold precursor was increased, more nuclei would form. This would deplete the amount of available reducing agent and would lead to very small particles with a very high gold content as the remaining silver could only marginally be reduced. The chosen molar gold content thus presents a compromise between the desired final composition of the particles, the desired final particle number concentration and the desired particle size.

**Fig. 6:**
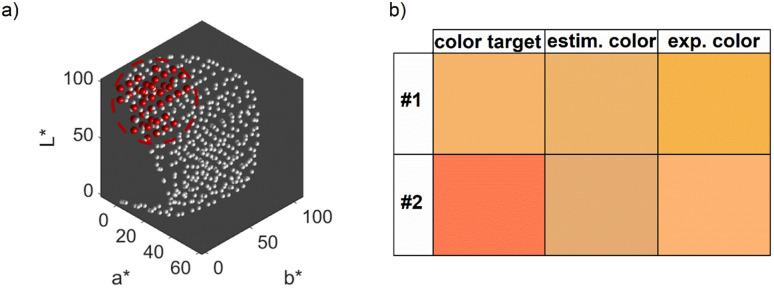
(a) Practically achievable color space of the chosen synthesis route. The spheres symbolize the entire theoretically available color space according to Mie simulations, while the red spheres are the colors that are also practically achievable by the synthesis (Euclidean distances to experimental values </= 5). (b) Visual comparison between the color targets, the predicted colors according to the FF model and the experimentally obtained colors at the optimal synthesis conditions.

Remarkably, at the optimum conditions shown in the bottom right sub-figure, an Euclidean distance to the color target of less than 1 is shown, which proofs excellent agreement between the desired color target and the practically achieved color at optimum conditions. This leads to the question of how the practically achievable color space of the chosen synthesis compares to the theoretically available color space shown in Fig. 2. We thus created a grid of more than 80 000 CIEL*a*b* coordinates within the available color space and used each of those points as a potential color target. Using our method, we determined the Euclidean distances between each of those grid points and each of the experimental data points. For distances smaller or equal to 5 we defined the color to be practically achievable. This gives us an effective method to determine the accessible color space of our synthesis route, which is otherwise hardly measurable. Fig. 6a shows a comparison between the theoretically available color space according to Mie calculations (all spheres – property function) and the practically available color space of our synthesis route according to the presented method (red spheres). It can be seen that only a very limited window of possible colors can actually be achieved using our synthesis route. Further it becomes evident that the first chosen color target (as discussed in Section 3.1 and Fig. 2) lies perfectly within the achievable color space, while the second color target is outside but within the theoretically available space.

Oftentimes the challenge in optimizing properties starts with the definition of a desired but not attainable property and lies within getting as closely as possible to this property. For our example this corresponds to finding the appropriate synthesis conditions to match the color of the second color target, which lies outside the practically achievable color space for our established synthesis route.

We therefore repeated the optimization procedure for the second color target and plotted the resulting 5-dimensional property–process relationship (see ESI Fig. S5†). The resulting trends are similar to those described for Fig. 5, with a slightly more pronounced increase in the optimal NaOH concentration between a metal precursor concentration of 0.875 mM and 1.25 mM, which is due to the lower L* value of color target #2. However, the Euclidean distances differ between the experimental data. While the distance at the global optimum for color target #1 is below 1, which is an almost perfect color fit, the distance for color target #2 is at values above 40, which shows a significant deviation. This is expectable, as we established, that the second target cannot be reached by the synthesis. Fig. 6b shows both color targets, the estimated optimum colors at the minimum of the optimization function and the experimentally achieved colors using the optimum process conditions. Visibly, it becomes clear that color target #1 is well matched experimentally, while there is a significant deviation for color target #2. Moreover, we see that the optimization function predicts the process conditions, which are needed to attain the predicted color accurately, as the visible difference between the second and third row is low. This is a first indication for the validity of the model, which will be discussed in detail in Section 3.2.4.

In summary, we successfully established a data-based property–process relationship, providing quantitative insights into the process conditions leading to a desired color. The trends observed can be explained by mechanistic aspects within the synthesis procedure, highlighting the value of the data-based model for better understanding of complex particle systems. We show that using our method, we can find synthesis conditions leading to an excellent property match if the chosen property target lies within the achievable space. If a target outside this space is chosen, the method still delivers the best possible conditions to get as closely to the target as possible. Further, we successfully used our method to determine the practically achievable property space of our synthesis route. This is extremely useful information for evaluating the use of a specific route for a specific optimization problem.

#### 3.2.4 Validation

**Fig. 7:**
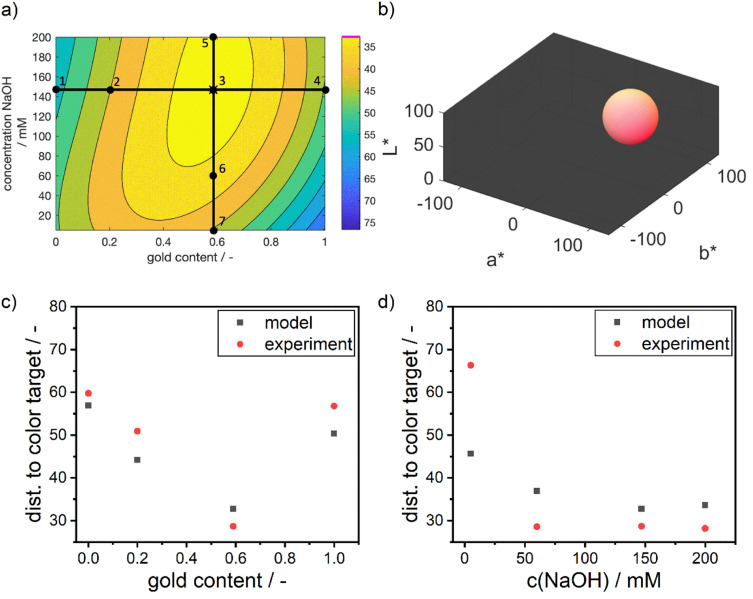
(a) Validation of data-based property–process relationship for silver–gold alloy NPs. The numbers 1 to 7 symbolize points at which the model was compared to experimental values. Point 3 is the global optimum found *via* the full-factorial approach. (b) Sphere of possible colors as received from Euclidean distance optimization. The colors shown on the outside of the sphere are the possible colors that can be found at the optimum without further information than the Euclidean distances to the color target. (c) Comparison between predicted distances to the color target *via* the data-based model and experiments at point 1 to 4 in (a). (d) Comparison between predicted distances to the color target *via* the data-based model and experiments at point 3 and 5 to 7 in (a).

To validate the obtained optimum, a series of validation experiments was conducted. Notably, the identified optimal process conditions differ from the experimental set used for model calibration. Therefore, congruence between the modeled data and the results of experimental validation serves as evidence of the capabilities of the model. To achieve this, multiple points (1–7) were selected across the property–process relationship map (refer to Fig. 7a), and the predicted values for the response variable, *i.e.*, the Euclidean distance between the resulting color and the color target were compared to experimental values (refer to Fig. 7c and d).

Visibly, the experimental distances align well with the predicted values in terms of absolute values and the described trends. As predicted by the model, the distance to the color target decreases from point 1 to the optimum at point 3, and then increases again towards point 4. Points 3 and 5 show nearly identical results, with point 5 being slightly worse than point 3. Points 6 and 7 exhibit the largest deviations between model and experiment, with point 7 showing a significantly larger deviation.

The increasing mismatch at low NaOH concentrations can be attributed to considerable deviations in the a* and b* values, while the L* value remains accurately predicted. This points towards potential discrepancies in the reaction chemistry or the stability of the particles. The accurate L* value indicates that the number of particles aligns with the predictions. However, upon examining the sample at point 7, a purple color is observed (see ESI Fig. S3†), suggesting the presence of larger and unstable particles with a high molar gold content. This instability may be due to the low NaOH concentration, leading to a reduced reductive capacity of the dextran reducing agent. As a result, the reduction rate is lower, leading to a lower supersaturation of silver and gold atoms in the synthesis solution, which in turn leads to the formation of large particles. These particles seem to be unstable under the given dextran concentrations, pushing the suspension to the edge of the stable parameter region and potentially causing agglomeration.

However, this stability issue is not fully captured by the model, as the resulting colors do not systematically vary with the process conditions. Instead, the model correctly suggests a red color for the suspension, indicating the formation of large and stable but highly gold-rich NPs due to the lack of reductive capacity to reduce the entire amount of silver present in solution. Thus, the model indicates a decreasing match in color compared to the defined color target (see Fig. 7d) but fails to predict the extent of the mismatch induced by the loss in NP stability.

#### 3.2.5 Additional process insights

While optimizing the process parameters based on the Euclidean distance in the CIEL*a*b* space alone results in a well-defined optimum in the parameters, it does not provide specific information about the exact color at the identified optimum. Consequently, the obtained optimum corresponds to a color situated at a certain distance from the color target, effectively forming a sphere in the 3-dimensional space with a radius equivalent to the optimized distance (refer to Fig. 7b). Depending on the radius of this sphere, the potential colors can exhibit considerable variation. To determine the color at the optimal parameter combination, conducting an additional lab experiment and recording the extinction spectrum would be necessary, which introduces extra experimental complexity and stress.

To address this concern, we decided to individually fit the L*, a*, and b* values across the entire parameter grid. This approach not only enables us to precisely determine the resulting color at the global optimum of the model but also provides valuable insights into the influence of process parameters on the color-defining values. In addition, for many applications color indicators such as hue, chroma or luminance are traditionally used to describe colors. Those indicators can be derived from the CIEL*a*b* coordinates (see eqn (12)–(14)), showing their universal applicability. As the CIEL*a*b* space is developed in accordance to human color perception, we chose to describe colors only in CIEL*a*b* coordinates.12
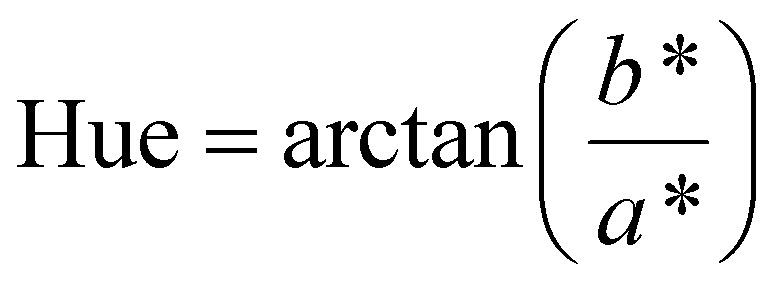
13
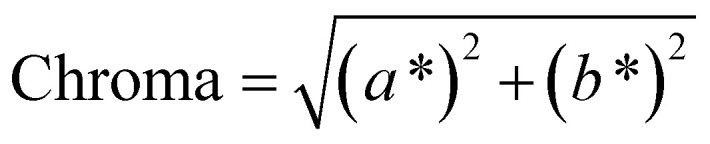

14 Luminance = L*

**Fig. 8:**
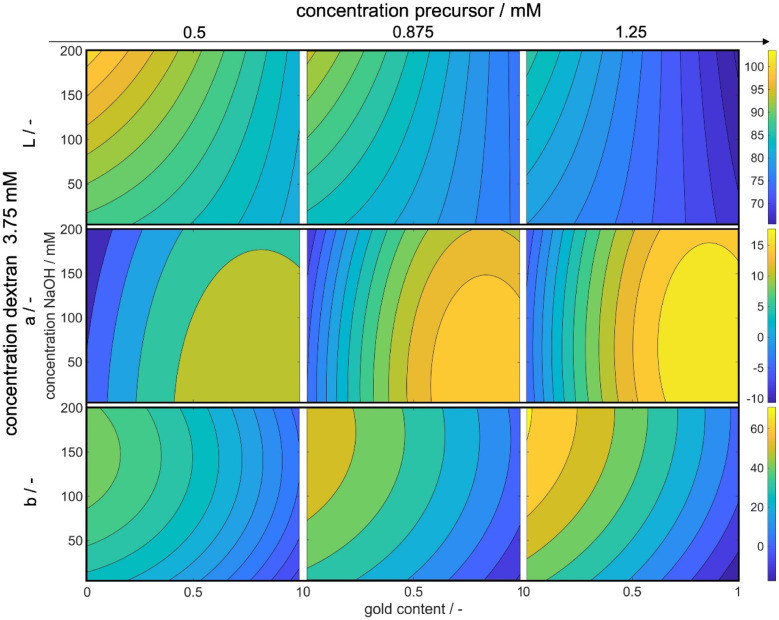
Influence of the process parameters on the color-defining L*, a* and b* values. The *x*-axis shows the molar gold content of the precursor solutions, while the *y*-axis shows the concentration of the added NaOH precursor. As the total parameter space for L*, a* and b* is 5-dimensional for every variable, 3 dimensions are shown in the figures, another is shown by an increase in the precursor concentration from the left to the right column and the 5^th^ dimension is set constant as a dextran concentration of 3.75 mM.

Fig. 8 illustrates the variations in the L*, a*, and b* values in dependence of the experimental process parameters. The *x*-axes represent the gold content of the metal precursor mixture, while the *y*-axes represent the concentration of added NaOH solution. The color bar indicates the absolute values of L*, a*, and b* respectively, with brighter colors indicating higher values. The complete parameter space for L*, a*, and b* is 5-dimensional for each variable. We display three dimensions in the figures while the fourth dimension is represented by an increase in the precursor concentration from the left to the right column. The fifth dimension is held constant at the optimal dextran concentration of 3.75 mM. We only show the maximum value in dextran concentration, as the influence of the reducing agent concentration on L*, a*, and b* values is small, while the trends are similar for all concentrations.

With increasing precursor concentration, the pH of the solution and the added NaOH concentration become less critical in achieving the correct L* value and, consequently, the desired color intensity. This behavior can be attributed to the formation mechanism of silver–gold alloy NPs. In our chemical co-reduction synthesis, both metal precursors come in contact before and during the particle formation. Solid AgCl precipitates due to its extremely low solubility at room temperature (Ag^+^ and Cl^−^ ions from silver and gold precursors, respectively). Therefore only the remaining gold nucleates. Over the process time, AgCl slowly dissolves, which leads to an increasing molar silver content in the particles over time and an increase in the core diameter. However, the size of the particles as shown in Section 3.1 only has a minimal impact on the resulting color. Instead, the particle concentration plays a crucial role as a result of the chosen metal precursor concentrations.

Moreover, the concentration of NaOH influences the reductive strength of dextran, resulting in a higher build-up of metal atom supersaturation and the formation of smaller NPs. The chosen NaOH concentration allows for the formation of gold nuclei even at low NaOH concentrations, making it less impactful on the particle concentration but more relevant to the particle size and chemical composition. The effect of NaOH concentration on color saturation becomes more pronounced with a decrease in metal precursor concentration, where particle size becomes more significant. Since the optimized color is a transmission color, its saturation is influenced by particle size, composition, and concentration. For lower concentrations, the effect of size and composition becomes more pronounced. Particularly at a molar gold content of 0% and the highest tested NaOH concentration, the color saturation reaches its lowest point. The red color of gold NPs exhibits a higher L* value than the yellow color of silver NPs, and thus, saturation is strongly dependent on the molar gold content, which can also be seen at high metal concentrations.

Furthermore, it is known that an increase in solution pH accelerates the reduction rate of silver but slows down the formation rate of gold. Therefore, low gold concentrations at high solution pH yield very small particles with low color saturation, resulting in high L* values. Notably, a reduced dextran concentration alters the trends slightly, mainly due to the pH-dependent reductive strength of dextran. At high dextran concentrations, the reductive strength is sufficient to achieve the desired color saturation, while at low dextran concentrations, a higher NaOH amount is needed to achieve the same effect (see ESI Fig. S2†).

Examining the a* values reveals a clear trend from lower to higher values with an increase in both the precursor concentration and the molar gold content. In the CIEL*a*b* space, increasing values of a* correspond to a shift from green to red.^51^ The resulting color of the particle suspensions shifts with increasing molar gold content from bright yellow to bright red due to changes in the position of the localized surface plasmon resonance.^13^ Consequently, an increase in the molar gold content leads to a simultaneous increase in the a* value.

The increase in the red value with higher metal precursor concentrations can be attributed to the scaled extinction band at elevated particle concentrations. An extinction band recorded at lower particle concentrations with defined height and width will expand in both dimensions at higher particle concentrations. Consequently, a broader extinction band, absorbing more light at higher wavelengths, causes the particle suspension to appear more red, resulting in an increased a* value.

Moreover, an increase in the precursor concentration requires a higher amount of NaOH solution to achieve the maximum a* value. This can again be explained by the pH-dependent reduction potential of the reducing agent dextran. With an increase in the gold precursor, the solution pH decreases, affecting the reductive potential of dextran. Additionally, more reductively active groups are required to reduce the added metal. Therefore, the addition of more NaOH serves two purposes: first, it neutralizes the reduction of pH caused by the gold precursor, and second, it increases the number of reductively active groups in the dextran molecules to facilitate the reduction process.

The b* value shows similar correlations, where an increase in the precursor concentration leads to an increase in the b* value, as explained earlier. However, in contrast to the behavior of the a* value, a decrease in the molar gold content of the particles results in an increase in the b* value. This is expected since the b* value describes a shift from blue to yellow colors,^51^ and a solution with lower gold content and higher silver content appears increasingly yellow.

Examining the dependency of the a* and b* values on the NaOH concentration and thus solution pH, it becomes evident that the b* value is more strongly affected compared to the a* value, especially at higher precursor concentrations. This behavior can be explained by the formation mechanism of the NPs. As previously described, during the initial stages of the process, particles with close to 100% molar gold content are formed due to the precipitation of AgCl and the higher reduction potential of gold ions.^52,53^ Subsequently, the amount of reduced silver ions depends on the reductive strength of the reducing agent. Thus, an increase in the NaOH concentration leads to more reduction of silver ions, resulting in an increase in the yellow color component and consequently, an increased b* value.

Conversely, the a* value, representing the red color component, is hardly affected, as the gold particles formed in the initial stages of the process already contribute significantly to the observed red color.

In conclusion, our model demonstrates a remarkable accuracy in describing the selected synthesis, successfully estimating even subtle changes in the desired property, namely the color of the final suspensions, under varying process conditions, based on a limited data set. Moreover, the model effectively captures dependencies between parameters, providing valuable insights for scientists seeking to comprehend the underlying formation processes and guiding them in conducting targeted experiments. The observed changes in suspension properties can be conclusively explained by physical phenomena, further validating the efficacy of our data-driven model. With these results, we have established a robust property–process relationship for the precise color design of silver–gold alloy NPs.

#### 3.2.6 Reduction of experimental work (DoE)

The derived property–process relationship is derived from a full-factorial (FF) experimental plan comprising a relatively small set of 81 experiments. Our goal is to achieve comparable process relationships as illustrated in Fig. 5 while further minimizing the experimental effort. To achieve this, we have adopted a design of experiment (DoE) approach. To reduce the number of required experiments further, while maintaining the accuracy and reliability of the property–process relationship, we have implemented both the Box–Behnken (BB) and the face centered central composite design (CCD), both capable of capturing quadratic dependencies.

**Fig. 9:**
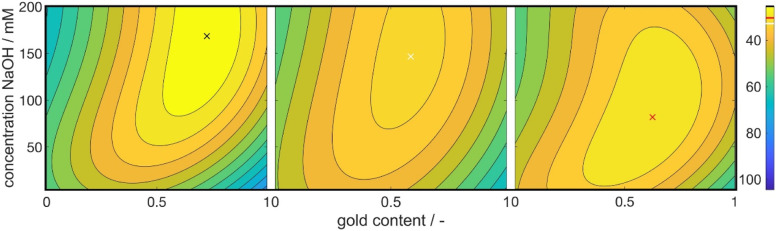
Optimal synthesis conditions to achieve the chosen color target as retrieved *via* the BB (left), FF (middle) and CCD design (right) respectively. The *y*-axis shows the NaOH concentration and the *x*-axis shows the intended gold content. Colors indicate the Euclidean distance to the color target in the CIEL*a*b* space. The exact values for the distances are shown in the color bars on the right with the colored line showing the position of the local optimum within the subplot as marked by the cross in the respective color.

Fig. 9 illustrates the global optima obtained from the three experimental approaches: full factorial (FF), Box–Behnken (BB), and face centered central composite design (CCD). The complete property–process relationship maps can be found in the ESI (see Fig. S5–S7†). While the FF plan was based on 81 experiments, the BB and CCD plans required only 27 and 36 experiments, respectively, resulting in a 2–3 fold reduction in experimental workload. Notably, the BB and CCD designs yielded higher *R*^2^ values for L*, a*, and b* compared to the FF design presented in Section 3.2. Specifically, the *R*^2^ values for the BB design were 83%, 94%, and 95%, while the CCD design achieved *R*^2^ values of 85%, 95%, and 91%, respectively. This improvement in correlation is expected, as the reduced number of measurement points in BB and CCD designs allows for a better fit of the chosen correlation function (refer to eqn (11)) to the data points. For any given fit function the *R*^2^ value decreases with an increase in points to be hit by the function (see Fig. S4†). Notably, a better fit does not relate to a more accurate or reliable property–process relationship.

Upon analyzing the optimal positions and overall trends depicted in the three diagrams, it becomes evident that all three experimental procedures successfully identify the same relationships between the investigated process parameters and the response variable. Moreover, the positions of the optimum in the 5-dimensional parameter space are very similar among the three plans. Table 2 provides an overview of the optimal process condition values obtained from the FF, BB, and CCD experimental plans.

**Table 2:** Optimum process conditions found for the full-factorial-, the Box–Behnken- and the face centered central composite design

Parameter/DoE model	FF	BB	CCD
*c*(dextran)/mM	3.75	3.75	3.75
*c*(metal precursor)/mM	1.25	1.25	1.25
Molar gold content/—	0.6	0.7	0.6
*c*(NaOH)/mM	147	168	82

The optimal conditions identified in all three experimental plans exhibit good agreement concerning the concentration of the reducing agent, metal precursor, and the intended molar gold content. However, a discrepancy is observed in the optimum NaOH concentration within the CCD plan, which is notably lower compared to the other experimental plans. This deviation may be attributed to the positioning of the grid points in the CCD plan, as discussed in Section 2.2. The central composite design aims to cover the available parameter space using a two-level design supplemented with face-centered points. Multivariate models are inherently more accurate near their grid points. As a result, the optimal values for the concentrations of dextran and metal precursor, located at the maximum value of the parameter space, are precisely identified.

Although the 4-dimensional input parameter space cannot be visualized in 3 dimensions, one can identify the two maximum precursor concentrations situated at the corners of a cube, as illustrated in Fig. 1. We propose that the NaOH concentration lies along an edge of this hypothetical cube, which is not adequately represented due to the lack of grid points on the edges. Meanwhile, the molar gold content is positioned close to the center of one of the cube's faces, where another grid point is located. Consequently, while the concentrations of dextran and metal precursor align well with measurement points, the molar gold content is not an exact match but still reasonably close to one of the grid points in the center of a face. Finally, the concentration of NaOH falls on an edge, *i.e.,* the model suggests that the optimum lies somewhere between the maximum and minimum values, but it fails to precisely predict the correct value.

In summary, all three experimental procedures reveal consistent property–process correlations. The estimated optimum conditions also exhibit good agreement across the protocols; however, certain variables show deviations depending on the position of the optimum value. Consequently, the strength of the resulting model decreases as the number of grid points decreases, but the key conclusions remain valid. Depending on the model's intended application, such as a guide for further elucidating experiments, the use of DoE methodologies has successfully reduced the required number of experiments by a factor of more than 3.

### 3.3 Evaluation of structure–property relationships

In Section 3.1, we discussed the optimization of the system's color using theoretical Mie calculations, assuming a monodisperse system. In contrast, Section 3.2 focused on optimizing the system's color based on the actual synthesis. While Section 3.1 emphasized the relationship between NP properties and the optical response of the particle suspension (structure–property relationship), Section 3.2 centered on the process parameters that yield the desired optical properties (property–process relationship). The chosen data-driven approach is therefore able to bridge the gap between property function and process function by providing a direct property–process relationship, it does not, however, directly provide information on the structure of the produced particles.

**Fig. 10:**
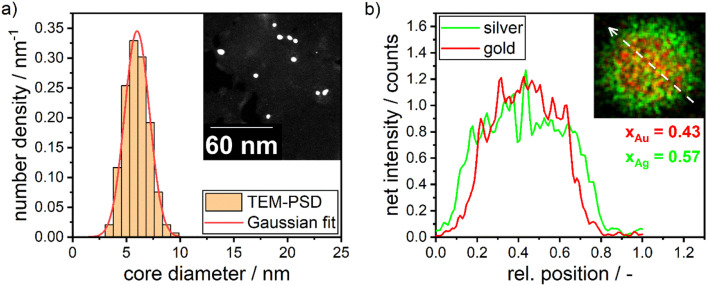
(a) PSD as retrieved from STEM images and the corresponding Gaussian fit. An example STEM image is shown in the inset. Around 200 particles have been counted to retrieve the distribution. (b) STEM-EDX maps (inset) and the corresponding silver and gold signal over the scanned diameter of the particle. The STEM-EDX maps show the net intensity of silver in green and gold in red, with the white arrow indicating the line scan.

In this section we therefore compare the predicted disperse properties of monodisperse particles with the particles produced in the synthesis at optimal synthesis conditions. Fig. 10 shows the disperse properties of the experimentally produced NPs according to color target #1, the particle size density distribution as retrieved from STEM images (Fig. 10a) and the STEM-EDX data (Fig. 10b). A normal distribution of core diameters with a mean value of 6.0 nm and a standard deviation of 1.3 nm is found. Additionally, when analyzing the molar gold content of the produced particles *via* STEM-EDX a value of 43% is retrieved, which is slightly, however not significantly lower than the 45% gold content fed into the experiment. This means that under the chosen process conditions not all of the gold precursor is reduced and/or crystallized into NPs. This can be explained by a change in the hydrochemistry of the system. As two metal precursors are present, both metals, their chemistry and consequently their reduction behavior is influenced differently by the process conditions, *i.e.*, here the pH of the reaction mixture. For high pH values, *i.e.*, under a large excess of OH^−^ ions, the reaction kinetics of silver ions to silver atoms is strongly enhanced,^49^ while gold forms complexes with the OH^−^ ions, which show a slower reaction kinetics.^20,54^

For high pH values this can lead to particles with a disproportionally higher silver content as well as more pronounced core–shell structures. This is a compromise between obtaining the correct size and particle number concentration on the one hand and achieving the best possible composition and homogeneous distribution of gold throughout the NP on the other hand. These factors are influenced by the solution pH. At high solution pH, the reductive capacity of the reducing agent increases, leading to a higher build-up of supersaturation and the formation of a larger number of smaller NPs. However, the high pH also alters the solution chemistry, resulting in changes in the composition and distribution of gold within the particles. A slight core–shell structure is visible in the STEM-EDX map in Fig. 10b, though not strongly pronounced as described in our recent publication.^13^ The silver enrichment on the surface of the particles can be explained by a variety of factors, including the lower surface energy of silver,^55^ the higher bond-strength of Au–Au bonds^55^ and the higher electrochemical potential of gold ions compared to silver ions.^56,57^ Additionally, as mentioned above, due to the high metal precursor concentrations, the NPs form as core–shell particles already during the synthesis, with the extent of the core–shell structure being influenced by the rate of silver atom formation. A respective study on the precise formation mechanism of the particles is currently being conducted within our group.

We can estimate the particle concentration yielded under optimal process conditions under the assumption of monodisperse NPs and the assumption of complete silver reduction. With the knowledge of the molar amount of silver in solution and the molar gold content of the final particles, the reduced molar amount of gold is given by:15
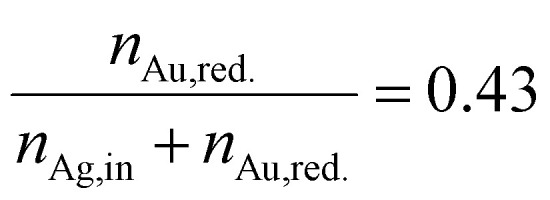
Here *n*_Au,red._ describes the reduced molar amount of gold and *n*_Ag,in_ describes the molar amount of silver fed into the synthesis mixture, which is assumed equivalent to the reduced molar amount of silver. The number of particles in one synthesis batch of 6 mL can then be calculated as follows:16
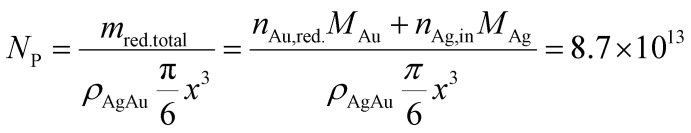
Here *m*_red.total_ is the total mass of reduced silver and gold, *ρ*_AgAu_ is the density of a silver–gold alloy NP with a molar gold content of 43% as measured, *M*_Ag_ and *M*_Au_ are the molar masses of silver and gold, respectively, and *x* is the mean core diameter of the final particles as measured. This yields a final particle number concentration of 1.5 × 10^13^ mL^−1^.

**Fig. 11:**
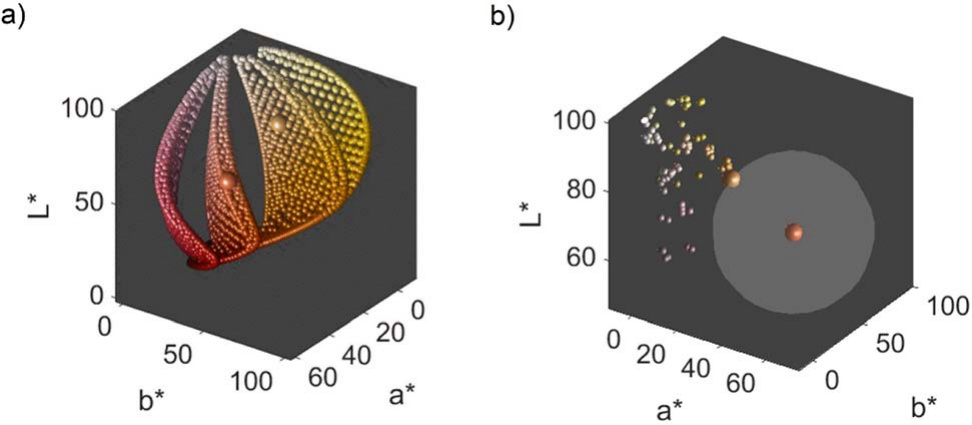
(a) Available colors from the available color space for 4 distinct molar gold contents of 100%, 83%, 50% and 0% represented by the 4 surfaces represented by the colored dots from left to right. The larger spheres show the color targets. (b) All colors produced within the process at different process conditions (small spheres) as well as the color at optimal conditions (larger sphere on the left) and its distance to the color target (larger sphere on the right). The grey sphere around the color target has the diameter of the minimum distance to the color target received at optimal process conditions found *via* the FF design for color target #2.

In comparison, the theoretical optimization yields a core particle diameter of 6.6 nm, a molar gold content of 50% (see Fig. 11a) and a particle concentration of 1.6 × 10^13^ mL^−1^ at the optimum shown in Fig. 3 (see Table 3). These data show excellent agreement in terms of the disperse properties of the final particles. This proofs the direct link between the particles' dispersity and their resulting optical properties (property function), which is also the basis of our data-based property–process relationship as the bridge between the property- and the process function.

**Table 3:** Comparison of optimum values for the particle number concentration, the mean core particle diameter and the molar gold content as found by theoretical Mie calculations and experimentally using a full-factorial (FF) experimental design. The optimum values are described by the smallest possible Euclidean distance to the defined color target

Parameter/model	Color target #1		Color target #2	
	Mie theory	FF	Mie theory	FF
Number concentration/mL^−1^	1.6 × 10^13^	1.5 × 10^13^	1.5 × 10^11^	3.4 × 10^13^
Particle diameter/nm	6.6	6.0	35.4	4.5
Molar gold content/—	0.50	0.43	0.83	0.45

Doing the same analyses for the particles produced at optimum conditions for color target #2 results in nanoparticles with a mean core diameter of 4.5 nm, a molar gold content of 45% and a particle number concentration of 3.4 × 10^13^ mL^−1^. Compared to the theoretically optimal values for this color target, predicting a mean core diameter of 35.4 nm, a molar gold content of 83% and a particle number concentration of 1.5 × 10^11^ mL^−1^, we observe a large difference between theoretical and experimental values (see Table 3). This is expected, as the target color is outside of the practically achievable color space for our synthesis route. However, even though color target #2 visually appears to be at least similar to the experimentally achieved color, the analysis of the NP dispersity shows tremendous differences. This shows that the disperse properties of NPs are extremely sensitive to changes in the resulting color. As the color is directly coupled to the disperse properties of the NPs *via* their property function (as discussed in Section 3.1), this means that the optimal disperse properties found *via* the Mie calculations will deviate strongly from those found at the optimum of the optimization function for the experimental values. Therefore, in order to get a well matching color, the disperse properties of the NPs need to match the theoretically predicted properties well. The differences thus clearly stem from experimental limitations as the desired color cannot be achieved through the selected process, even though it lies within the theoretical color space (refer to Fig. 6a and 11b). Nonetheless, we can clearly show that our method is capable of optimizing the synthesis conditions for finding a color, which is as close as possible to the desired but un-achievable color target. This can be seen in Fig. 11b, where all experimental data points are shown as small spheres. From these data points, the optimum synthesis conditions were determined. The resulting color at these optimal conditions is shown as a larger sphere on the left. The color target is shown as the larger sphere on the right. It can clearly be seen that the color found at optimal conditions is significantly closer to the target than the rest of the experimental points. Notably, the optimal conditions are not part of the training data set and thus amount for a completely new combination of parameters found by the trained model.

Overall, our study demonstrates that the process conditions identified through our data-based property–process relationship result in optimal particle structures, as predicted by the theoretical structure–property relationship. This finding provides strong evidence for the physical validity of our model and highlights the effectiveness of data-based property–process relationships in dealing with highly complex particle systems.

## 4 Conclusion

This article presents a comprehensive study on the targeted color design of silver–gold alloy NPs through a multivariate optimization approach. The synthesis of NPs is known to involve interconnected parameters that cannot be independently varied, and as such, we employed a data-based property–process relationship to optimize the optical properties of the NPs effectively. We defined a color target and utilizing a green chemical co-reduction method at room temperature, optimized the process parameters to achieve a color closest to this defined target. The optimization process was performed in the CIEL*a*b* color space, utilizing Euclidean distances as a metric for color matching. Our approach involved fitting a quadratic function to a defined grid of points within the parameter space to establish the property–process relationship. Simultaneously, we conducted theoretical Mie calculations to establish a structure–property relationship, considering a range of particle sizes, concentrations, and molar gold contents.

The results revealed distinct optima in both the theoretical structure–property relationship and the process–property relationship. Comparison between the theoretically optimal structure from Mie modeling and the experimental particle structures at the optimum of the property–process relationship demonstrated good agreement in terms of particle size, concentration and gold content. However, even a slight mismatch in the visually perceived color can lead to large differences in the disperse properties of the resulting particles, proofing strong sensitivity on the NP structure. The data-driven property–process relationship not only provides predictive insights into ideal process parameters of complex particle systems, but also offers valuable information on physical correlations occurring during the synthesis process as well as a way to identify the accessible property space for a given synthesis route. Moreover, it offers valuable insights into how the color of the NPs changes with varying process parameters.

To validate the model, we tested it on points outside of the original grid-points used for training, and it consistently predicted correct trends in and in most cases even quantitatively correct values for colors. Additionally, by finding the optimum of the fitted model, it yielded optimum process conditions producing colors much closer to the target than any of the original grid-points, indicating the model's accuracy and effectiveness. Furthermore, we found that employing Design of Experiments (DoE) methods could reduce the required experimental work by a factor of three. Although there may be a slight loss of accuracy with this approach, the efficiency gained justifies its adoption for practical applications.

In conclusion, our study offers a novel and effective methodology for the targeted color design of silver–gold alloy NPs. It demonstrates the importance of data-based property–process relationships in optimizing the optical properties of complex NP systems with limited information on the underlying formation mechanism and provides valuable insights for researchers in tailoring NP characteristics for specific applications in various fields, such as optoelectronics, catalysis, and biomedicine. The presented approach is not restricted to the particular synthesis presented here but can be used to optimize various particle properties within various formation processes. Examples could be, for instance, dewetting processes or the synthesis of ternary alloy systems and alloys with different valences. Even applications in catalysis might be possible: what is required is firstly a physical relationship between the disperse properties of the synthesized particles and the property to optimize (property function) and secondly that the disperse properties of the synthesized particles can be traced back to a change in the chosen process conditions (process function). This research constitutes a significant contribution to the field and paves the way for further advancements in NP synthesis and design.

Moving forward, our focus lies in broadening the scope of the model's utility by applying it to alternative synthesis approaches and a wide array of particle systems encompassing various experimental timeframes. The potential of data-driven models for both structure–property and property–process correlations is immense, offering a means to bridge gaps in the physical modelling of particle attributes based on their structures. This allows for the optimization of particle systems not solely based on properties that are physically well understood (*e.g.*, solid–light interaction), but also considering additional aspects such as catalytic activity, biocompatibility, and more.

## Data availability

Data for this paper, including all data included in the figures are available at https://Zenodo.org at https://doi.org/10.5281/zenodo.8355470.

## Conflicts of interest

There are no conflicts to declare.

## Supplementary Material

NA-006-D3NA00856H-s001
