# Supplementary material for: Targeted color design of silver–gold alloy nanoparticles
Source: Nanoscale Adv. 2023 Dec 7;6(5):1392–408. doi: 10.1039/d3na00856h (PMC10898422; doi:10.1039/d3na00856h)
Supplement: NA-006-D3NA00856H-s001 [file NA-006-D3NA00856H-s001.pdf]

Electronic Supplementary Material (ESI) for Nanoscale Advances.
This journal is © The Royal Society of Chemistry 2023

Electronic Supporting Information
Targeted color design of silver-gold alloy nanoparticles

N. E. Traoré [a,b], C. Spruck [a], A. Uihlein [c], L. Pflug [d], W. Peukert [a,b]

[a)] Institute of Particle Technology, Friedrich-Alexander-Universität Erlangen-Nürnberg, Cauerstraße 4, 91058 Erlangen, Germany

[b)] Interdisciplinary Center for Functional Particle Systems, Friedrich-Alexander-Universität Erlangen-Nürnberg, Haberstraße 9a, 91058 Erlangen, Germany

[c)] Department of Mathematics, Chair of Applied Mathematics (Continuous Optimization), Friedrich-Alexander-Universität Erlangen-Nürnberg, Cauerstraße 11, 91058 Erlangen, Germany

[d)] Competence Unit for Scientific Computing (CSC), Friedrich-Alexander-Universität Erlangen-Nürnberg (FAU), Martensstraße 5a, 91058 Erlangen, Germany

*Corresponding author: W. Peukert: wolfgang.peukert@fau.de

Synthesis of silver-gold alloy nanoparticles at high precursor concentrations

As described in the manuscript, the optimized silver-gold (AgAu) alloy nanoparticles (NP) are highly efficient absorbers. Thus, for high particle concentrations the resulting color of the NP suspensions is ill-defined, i.e., dark brown to black (see Figure S 1). The shown particle suspensions have a particle concentration of 25 mgL^{-1}, 50.8 mgL^{-1}, 76.2 mgL^{-1} and 101.6 mgL^{-1} from left to right at a molar gold content of 50 %. As can be seen, the first sample shows a distinct orange color, while the samples at higher concentrations show increasingly dark and ill-defined colors.

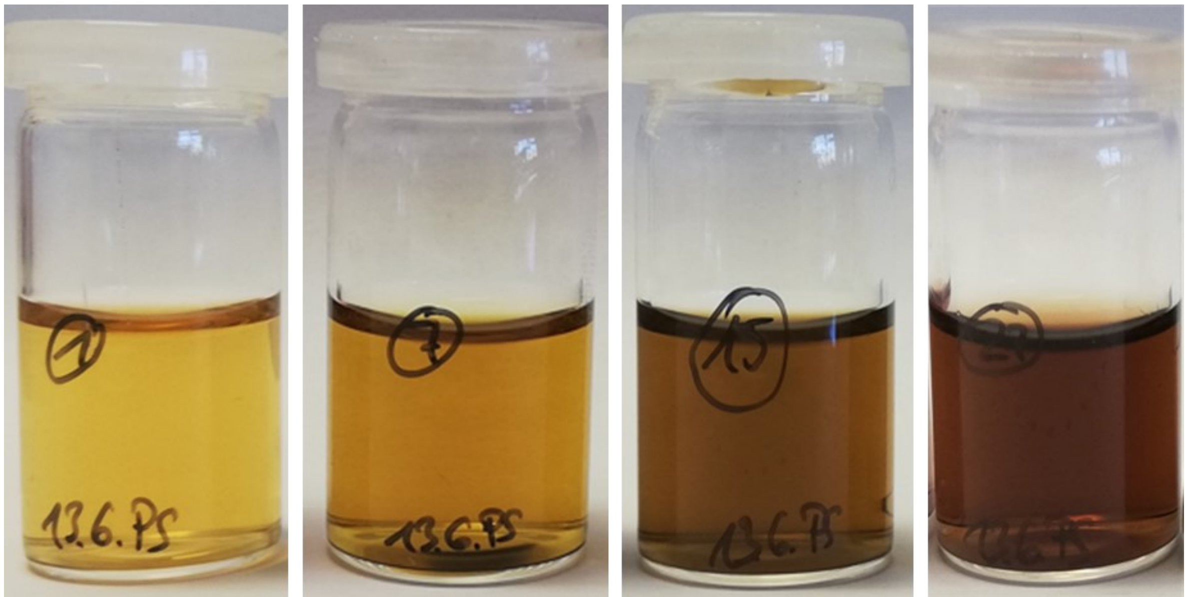

Figure S 1: AgAu alloy NP suspensions with a molar gold content of 50 % and NP concentrations of 25 mgL^{-1}, 50.8 mgL^{-1}, 76.2 mgL^{-1} and 101.6 mgL^{-1} from left to right respectively.

Correlation functions

A quadratic correlation function was chosen to fit the L*, a* and b* coordinates at different experimental conditions (see equation 10 in the manuscript). The values of the determined pre-factors $i_1, \ldots, i_{15}$ can be found in Table S 1.

Table S 1: Determined pre-factors of the quadratic correlation functions for L, a* and b*.*

	**L***	**a***	**b***
i_1	91.8650	-6.0346	5.1590
i_2	0.0257	0.0198	0.1048
i_3	-3.9257	2.0722	4.1731
i_4	0.8384	-4.4154	33.9271
i_5	17.9900	36.2876	20.1081
i_6	$-3.2612 \cdot 10^{-6}$	$-1.2215 \cdot 10^{-4}$	$-7.0628 \cdot 10^{-4}$
i_7	0.0140	-0.0106	0.0151
i_8	1.0586	-0.4113	-0.6218
i_9	-0.0333	0.0286	0.0985
i_{10}	-1.2545	1.0665	-0.0166
i_{11}	-4.6964	0.4027	-7.6922
i_{12}	-0.0514	0.0011	-0.0145
i_{13}	-5.5150	2.1748	-3.9618
i_{14}	-4.2877	3.4878	-34.3783
i_{15}	-3.1665	-28.3142	-23.4811

Effect of dextran concentration on color coordinates

Dextran was chosen as the reducing agent as well as the stabilizer in the described synthesis of AgAu alloy NPs. Changing the dextran concentration has an impact on the NP suspensions and the resulting colors. Figure S 2 shows the variations in L* in dependence of the dextran concentration in detail. In general, the correlations of the L*, a* and b* color coordinates with the dextran concentration show no profound effect. However, the trends described in the manuscript for L* are shifted for lower dextran concentrations.

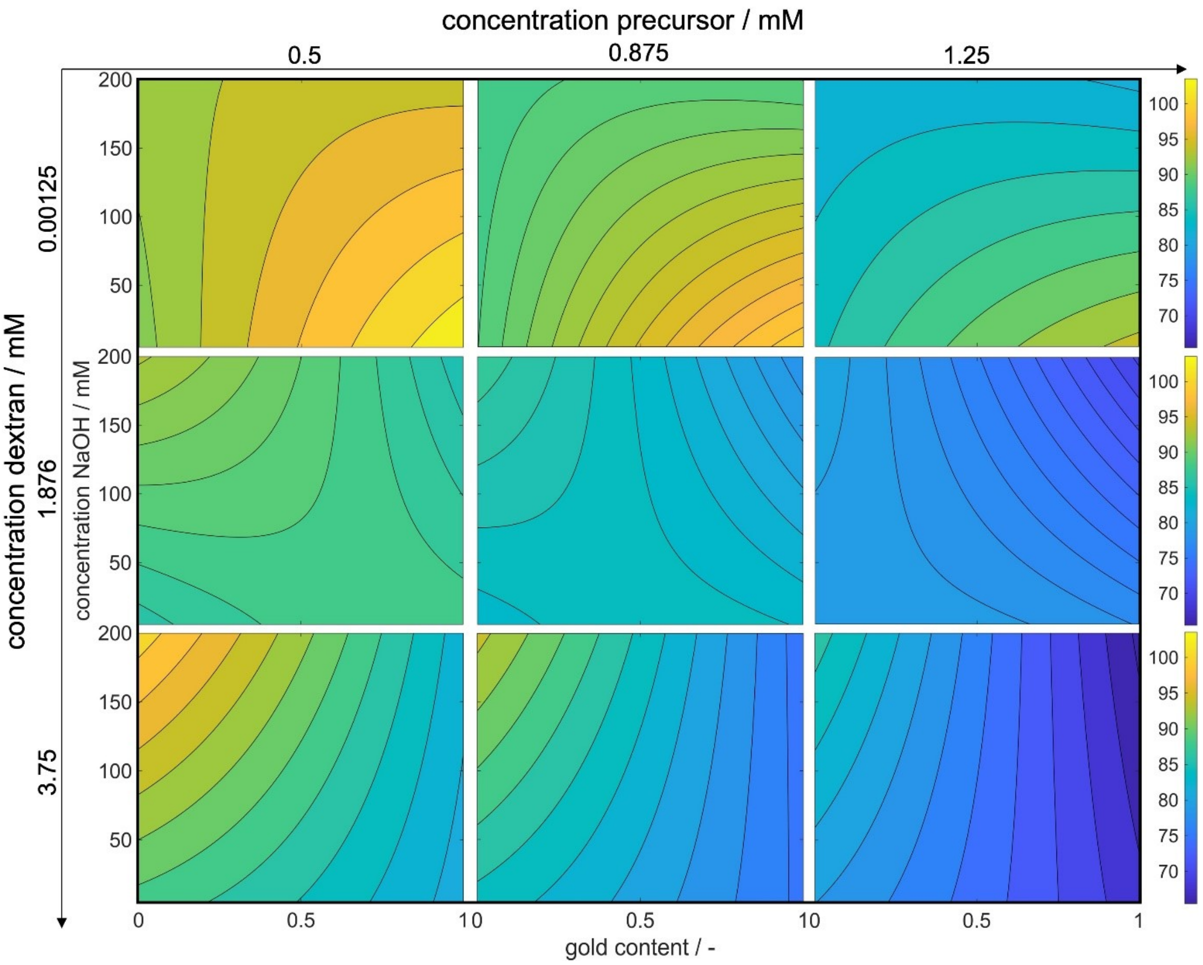

Figure S 2: Influence of the process parameters on the luminance defining L value. The x-axis shows the molar gold content of the precursor solutions, while the y-axes show the concentration of the added NaOH precursor. As the total parameter space is 5-dimensional, 3 dimensions are shown in the figures, another is shown by an increase in the precursor concentration from the left to the right column and the 5th dimension is shown by an increase in the dextran concentration from the top to the bottom row.*

Nanoparticle stability

An increasing mismatch at low NaOH concentrations between the predicted Euclidean distances to a defined color target and the equivalent experimental values were observed. This mismatch can be attributed to decreasing particle stability at these conditions. Figure S 3 shows the extinction spectrum as well as a photo of the corresponding sample (inset) for a metal precursor concentration of 1.25 mM, a dextran concentration of 3.75 mM, a molar gold content of 0.59 and a NaOH concentration of 5 mM, corresponding to point 7 in figure 7 of the manuscript. It can be seen that the sample is purple in color, suggesting the presence of larger and unstable particles with a high molar gold content. An extinction value above 0 at high wavelengths and a localized surface plasmon resonance (LSPR) position above 520 nm, which is a typical value for pure gold NPs, support this assumption.

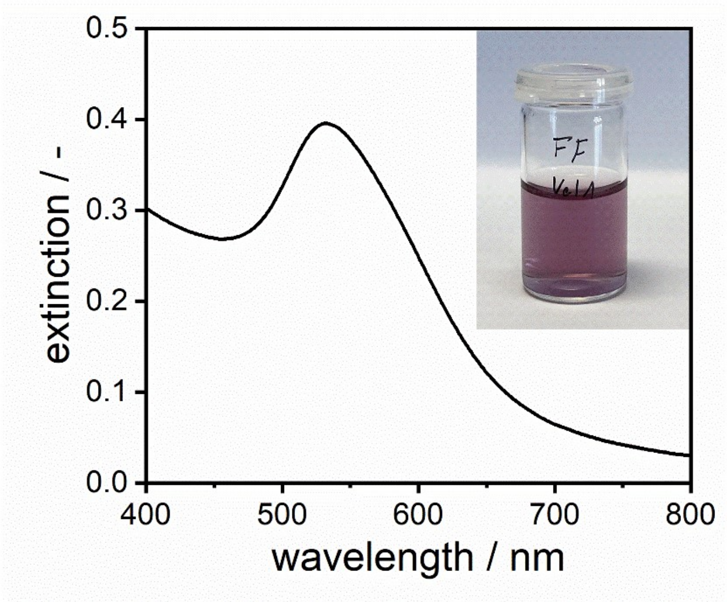

Figure S 3: Extinction spectrum and photo (inset) of an AgAu alloy NP sample corresponding to point 7 in figure 7 of the manuscript. The color and the spectrum indicate the presence of larger, partially instable NPs with a high molar gold content.

Quality of mathematical fits for increasing number of points

It was observed that the achieved R^2 values for L*, a* and b* is higher for experiments conducted according to the BB and CCD approach. This can be explained by the reduced number of measurement points that needs to be hit by the correlation function. For any given function the R^2 value decreases with an increase in the number of points that need to be hit by the function. Figure S 4 shows that for a 4th order polynomial, fit to an increasing number of random points (x-axis). As described, the R^2 value (y-axis) decreases with an increase in random points.

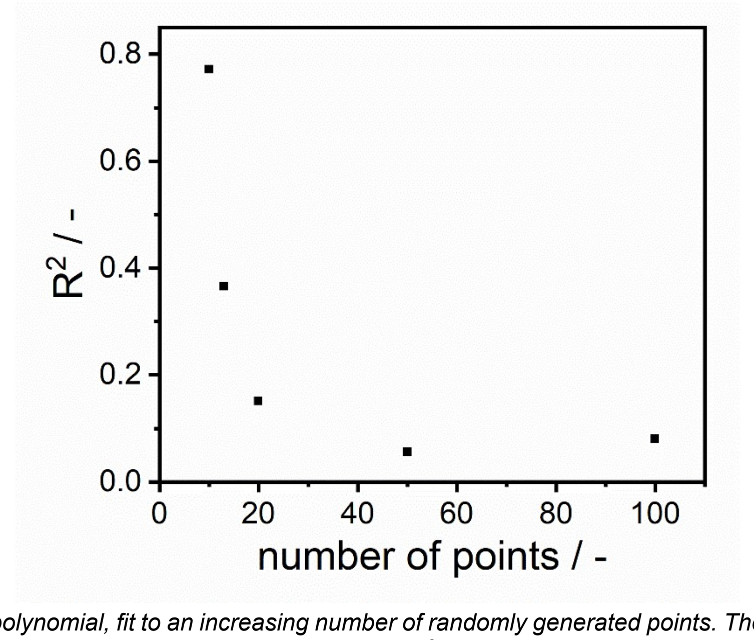

Figure S 4: 4th order polynomial, fit to an increasing number of randomly generated points. The number of randomly generated points is plotted on the x-axis, while the resulting R^2 value is plotted on the y-axis.

Experimental property-process relationships

After proofing our method will result in the correct color value if the color target is within the practically available region, we repeated our optimization for a desirable color target outside of the practically available but inside the theoretically available color space according to Mie calculations. Figure S 5 shows the property-process maps for this color target #2. Then, to reduce the experimental effort for determining data-based property-process relationships compared to the full-factorial approach, we employed the Box-Behnken (BB) and the Face Centered Central Composite (CCD) design. As shown in the manuscript, the general trends were identical while decreasing the number of experiments by a factor of 2-3. Figure S 6 shows the full property-process map for the BB and Figure S 7 for the CCD.

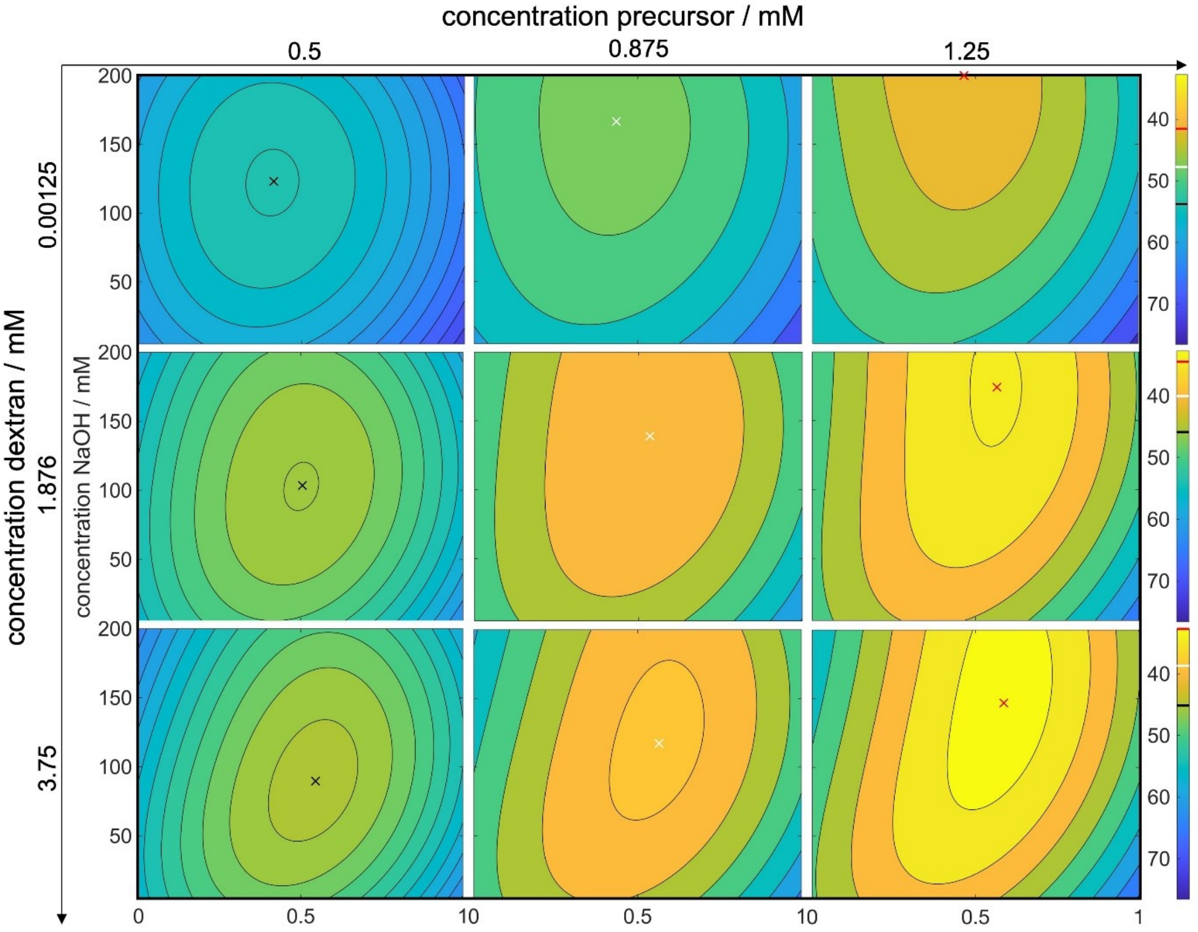

*Figure S 5: 3-dimensional plots of the Euclidean distances between the calculated CIEL*a*b* coordinates and the defined color target. Bright values symbolize optimal conditions, while darker colors symbolize larger distances to the color target. The exact values for the distances are shown in the color bars on the right of each line with the colored line showing the position of the local optimum within the subplot as marked by the cross in the respective color. The y-axes show the concentration of NaOH and the x-axes show the molar gold content. The concentration of metal precursor increases from the left to the right column of subplots, while the concentration of dextran increases from the top to the bottom row. The experiments used to retrieve the presented property-process map were designed via the Full-factorial design.*

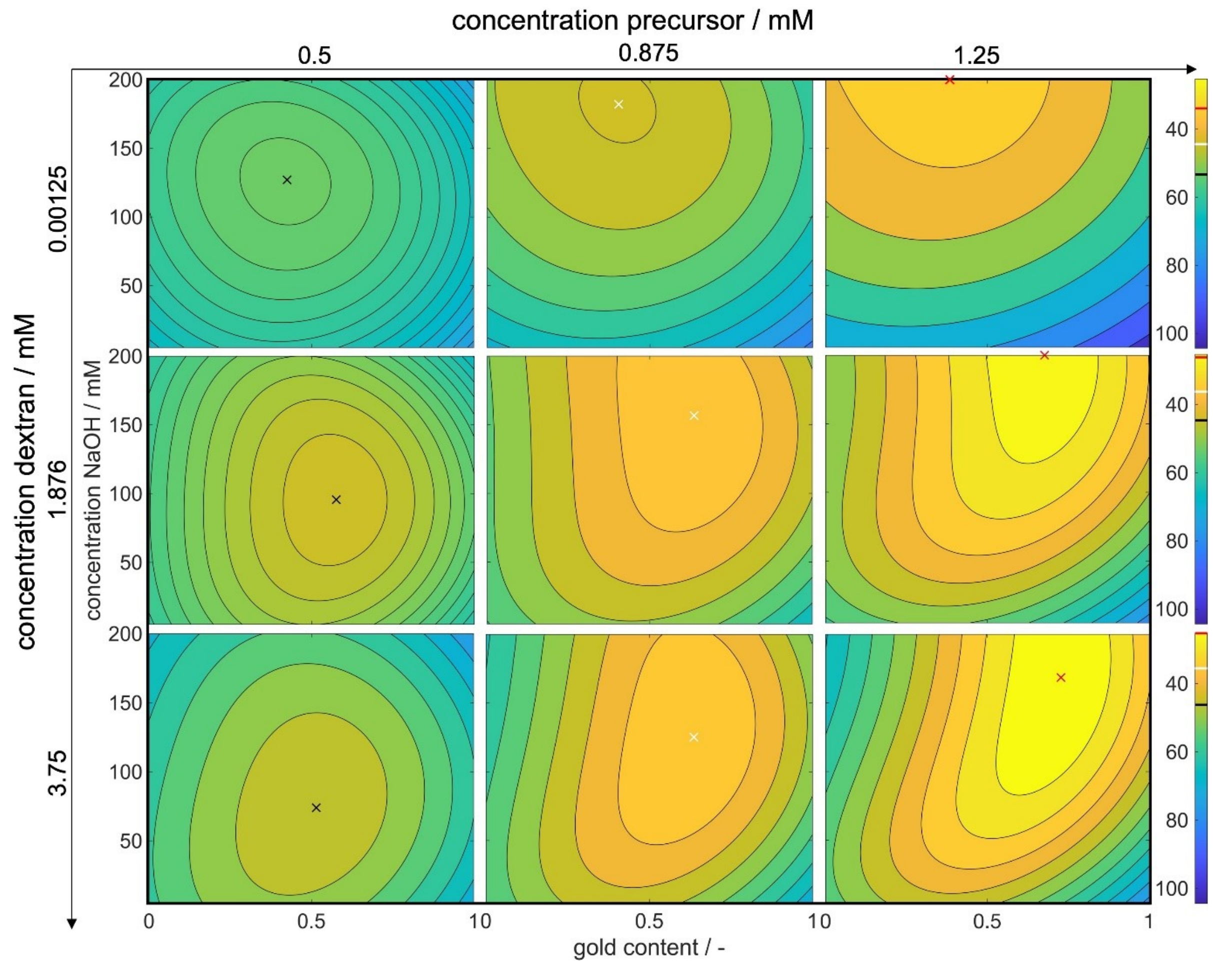

*Figure S 6: 3-dimensional plots of the Euclidean distances between the calculated CIEL*a*b* coordinates and the defined color target. Bright values symbolize optimal conditions, while darker colors symbolize larger distances to the color target. The exact values for the distances are shown in the color bars on the right of each line with the colored line showing the position of the local optimum within the subplot as marked by the cross in the respective color. The y-axes show the concentration of NaOH and the x-axes show the molar gold content. The concentration of metal precursor increases from the left to the right column of subplots, while the concentration of dextran increases from the top to the bottom row. The experiments used to retrieve the presented property-process map were designed via the Box-Behnken design.*

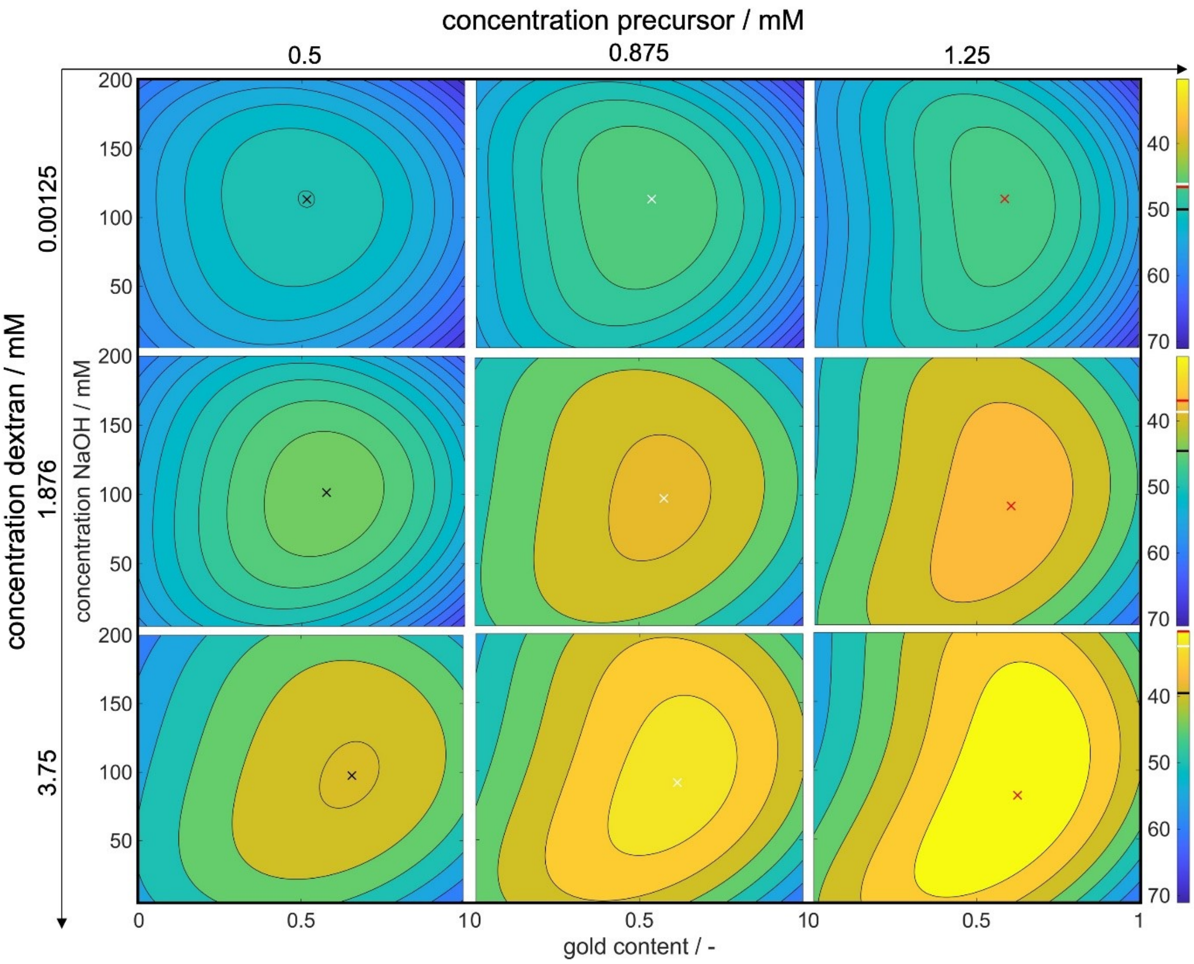

*Figure S 7: 3-dimensional plots of the Euclidean distances between the calculated CIEL*a*b* coordinates and the defined color target. Bright values symbolize optimal conditions, while darker colors symbolize larger distances to the color target. The exact values for the distances are shown in the color bars on the right of each line with the colored line showing the position of the local optimum within the subplot as marked by the cross in the respective color. The y-axes show the concentration of NaOH and the x-axes show the molar gold content. The concentration of metal precursor increases from the left to the right column of subplots, while the concentration of dextran increases from the top to the bottom row. The experiments used to retrieve the presented property-process map were designed via the face centered central composite design.*
